# Early progressive ambulation in neurosurgical patients with tracheostomy: clinical application and evaluation

**DOI:** 10.3389/fneur.2025.1591273

**Published:** 2025-06-09

**Authors:** Xiangyi Yin, Lihui Zhou, Yan Jia, Xiaowen Zhu

**Affiliations:** ^1^Department of Neurosurgery, The Affiliated Suzhou Hospital of Nanjing University Medical School, Suzhou, China; ^2^Department of Surgery, The Affiliated Suzhou Hospital of Nanjing University Medical School, Suzhou, China; ^3^Department of Nursing, The Affiliated Suzhou Hospital of Nanjing University Medical School, Suzhou, China

**Keywords:** ambulation, neurosurgical, tracheostomy, nursing, care, rehabilitation, treatment

## Abstract

**Background:**

Tracheostomy is a common procedure among neurosurgical patients, and preventing pulmonary infection while accelerating recovery is crucial for improving their clinical outcomes.

**Objectives:**

The objective of this study was to systematically assess the efficacy and safety of early progressive ambulation in neurosurgical patients who have undergone tracheostomy, to provide evidence-based insights to guide clinical treatment and nursing practices.

**Methods:**

Neurosurgical patients undergoing tracheostomy at our hospital between September 1, 2023, and January 31, 2025 were included. Patients were randomly assigned to either the progressive ambulation group or the routine care group. We established a multidisciplinary team (MDT) led by neurosurgical nurses to develop and implement an early progressive ambulation protocol for neurosurgical patients.

**Results:**

A total of 59 neurosurgical patients who underwent tracheostomy were enrolled, with 29 patients assigned to the progressive ambulation group and 30 patients to the standard routine nursing care group. The findings demonstrate that progressive ambulation offers substantial clinical benefits for neurosurgical patients following tracheostomy. Specifically, this intervention significantly reduces the incidence of pulmonary infections, decreases antimicrobial use density (AUD), and shortens the duration of antibiotic therapy. Furthermore, it is associated with a reduction in the duration of tracheostomy dependency and overall hospital stay, as well as a notable decrease in hospitalization costs. However, no statistically significant difference in mortality was observed.

**Conclusion:**

These findings highlight the potential of progressive ambulation as a clinically valuable intervention for improving outcomes in neurosurgical patients undergoing tracheostomy. Further research is necessary to explore its broader applicability across diverse patient populations, identify optimal implementation strategies, and assess long-term effects.

## Introduction

Tracheotomy is a common surgical procedure widely used in critically ill patients with upper airway obstruction, those requiring prolonged mechanical ventilation, and those in need of respiratory support (1). Owing to its simplicity and low cost, tracheotomy has been extensively applied in clinical practice. It effectively relieves upper airway obstruction, alleviates dyspnea, reduces airway resistance, and decreases anatomical dead space (2). Additionally, tracheotomy can improve respiratory mechanics, enhance patient comfort, and facilitate the clearance of airway secretions (3). Epidemiological data indicate that the utilization rate of tracheotomy is increasing annually (4). In a study of patients with acute respiratory failure, the rate of tracheotomy reached 10.8% (5). Moreover, patients who underwent tracheotomy had longer hospital stays and significantly higher mortality rates compared to those who did not. Another study reported that approximately 6 to 11% of patients in the intensive care unit (ICU) ultimately required tracheotomy to establish an artificial airway (6). However, tracheotomy may also lead to a series of complications. The surgical incision disrupts the body’s natural defense barriers, particularly the mucosal barrier of the respiratory tract, thereby impairing the patient’s original airway protective mechanisms (7). Combined with factors such as prolonged bed rest and compromised immune function, patients are highly susceptible to pulmonary infections (8). Other complications following tracheotomy include incisional infection, granulation tissue formation, accidental decannulation, and pneumothorax (9). These complications not only prolong hospitalization but also increase the consumption of medical resources (10). Therefore, in clinical practice, it is crucial to appropriately determine the timing and indications for tracheotomy and optimize postoperative care and management. These measures are of significant importance for reducing the incidence of complications and improving patient outcomes.

Scientific and rational nursing interventions are crucial for reducing complications associated with tracheotomy and ensuring favorable outcomes in neurosurgical patients who have undergone tracheotomy. One of the key interventions is early progressive ambulation activities. These activities follow a stepwise approach, encompassing multiple stages such as sitting up in bed (with the back unsupported), sitting at the edge of the bed, sitting out of bed, standing at the bedside, and walking (11, 12). The ultimate goal is to achieve independent ambulation for the patient. However, current research on the safety and efficacy of early ambulation activities in neurosurgical tracheotomy patients is relatively limited both domestically and internationally, and there is a lack of accurate data on the rate of out-of-bed activities (13). This situation is primarily attributed to the following factors: First, tracheotomy patients typically present with severe physical trauma, multiple indwelling catheters, and general debilitation (14). Second, there is a relative shortage of clinical healthcare human resources. Additionally, adverse events that may occur during early out-of-bed activities, such as falls, unplanned tube dislodgement, and changes in clinical condition, further increase the risks and challenges associated with implementation (15). Despite these challenges, early out-of-bed activities hold significant importance for patient recovery. Studies (16, 17) have shown that early progressive out-of-bed activities can effectively enhance muscle strength, improve organ function, and promote the recovery of self-care abilities. They also accelerate the restoration of independent functional capabilities and boost patients’ confidence in returning to daily life and family, offering new hope for recovery. Therefore, early progressive ambulation activities are of vital importance for the prognosis of neurosurgical tracheotomy patients.

## Objectives

The present study aims to establish a multidisciplinary team (MDT) led by neurosurgical nurses to develop and implement an early progressive ambulation protocol for neurosurgical patients, to provide a scientific and reliable basis for the nursing and rehabilitation of tracheotomy patients in clinical practice.

## Methods

This study employed a randomized controlled trial (RCT) design. The study protocol was reviewed and approved by the hospital’s ethics committee (approval number: IRB2025032). Written informed consent was obtained from all participating patients and their family members prior to enrollment.

In our RCT, the sample size was calculated to ensure adequate statistical power to detect clinically significant differences between the intervention and control groups. The primary outcome was incidence of pulmonary infection in stroke patients, and the calculation was based on a significance level (*α*) of 0.05 and a power (1−*β*) of 80%. Using the formula for comparing two independent groups, ${\left(\frac{Z_{\alpha /2}+Z_{\beta }}{\Delta /\sigma }\right)}^{2}$ , where *Z*_*α*/2_ is the standard normal deviate corresponding to the significance level (1.96 for α = 0.05), *Z_β_* is the standard normal deviate corresponding to the desired power (0.842 for power = 80%), *Δ* is the expected mean difference between groups, and *σ* is the pooled standard deviation, we determined that a sample size of approximately 25 subjects per group, totaling 50 subjects for the entire study, would be required.

This study consecutively included adult stroke patients (age ≥18 years) who met the following inclusion criteria: (1) admission to the Neurosurgery Department of our institution between September 1, 2023 and January 31, 2025; (2) undergoing primary tracheostomy during the current hospitalization; (3) absence of active infection or suspected infection at the time of tracheostomy; and (4) anticipated hospitalization duration >5 days post-procedure.

Exclusion criteria comprised: (1) history of previous tracheostomy; (2) diagnosis of community-acquired pneumonia upon admission; (3) mortality within 5 days of hospitalization; and (4) incomplete clinical records. Patients who were transferred to other departments or healthcare facilities during the study period were considered withdrawn from the study.

Randomization was implemented using a computer-generated random sequence. Participants were assigned to either progressive ambulation group or the routine care group in a 1:1 ratio. The randomization sequence was generated by an independent statistician who was not involved in the recruitment or treatment of participants. This method ensured that the allocation was unbiased and maintained the integrity of the study design.

The indications for tracheotomy in our study were determined based on established clinical guidelines (4, 18) and the specific needs of our patient population. Tracheotomy was performed for patients who were anticipated to require mechanical ventilation for more than 5 days due to prolonged ventilator dependence. Additionally, it was indicated for patients with impaired neurological status or those at risk of aspiration to ensure airway protection. The procedure also facilitated improved airway management by allowing better control of airway secretions and reducing the risk of complications associated with prolonged endotracheal intubation.

The decision to remove the tracheal intubation was guided by several criteria. Patients were considered for decannulation once they demonstrated stable respiratory function without the need for mechanical ventilation, ensuring airway integrity with no obstructions or complications that would preclude safe removal of the tube. Furthermore, patients had to be able to protect their airway, manage secretions, and demonstrate adequate respiratory function to maintain oxygenation and ventilation. Regarding discharge status, not all patients were discharged immediately after tracheal intubation removal; some remained in the hospital for further medical management or rehabilitation. The decision to discharge was based on the overall clinical status and readiness for home care. For patients who were discharged with the tracheal intubation still in place, the duration of tracheal intubation was calculated from the time of insertion until the time of discharge. This approach ensured accurate tracking of the duration of intubation, even if the patient was still dependent on the tube at the time of discharge.

Patients in the control group received the standard post-tracheotomy care protocol in the neurosurgical department. This protocol included maintaining the ward environment at a temperature of 21–24°C and a humidity level of 60%. Early enteral nutrition was initiated, with appropriate nutritional fluids selected according to medical orders and administered continuously via a nutrient pump to ensure patency of the nasogastric tube. The tracheal tube was secured with an appropriate degree of tightness, defined as allowing a single transverse finger width between the securing strap and the neck. Nurses on each shift were required to promptly check the fixation status to prevent loosening and to monitor the skin condition around the tracheostomy site. The skin surrounding the tracheostomy tube was disinfected with 0.5% povidone-iodine to maintain cleanliness and dryness. Gauze dressings were routinely changed twice daily, with immediate replacement if heavily contaminated. Additionally, the inner cannula of the tracheostomy tube was disinfected twice daily as part of the standard care regimen.

**Figure 1:**
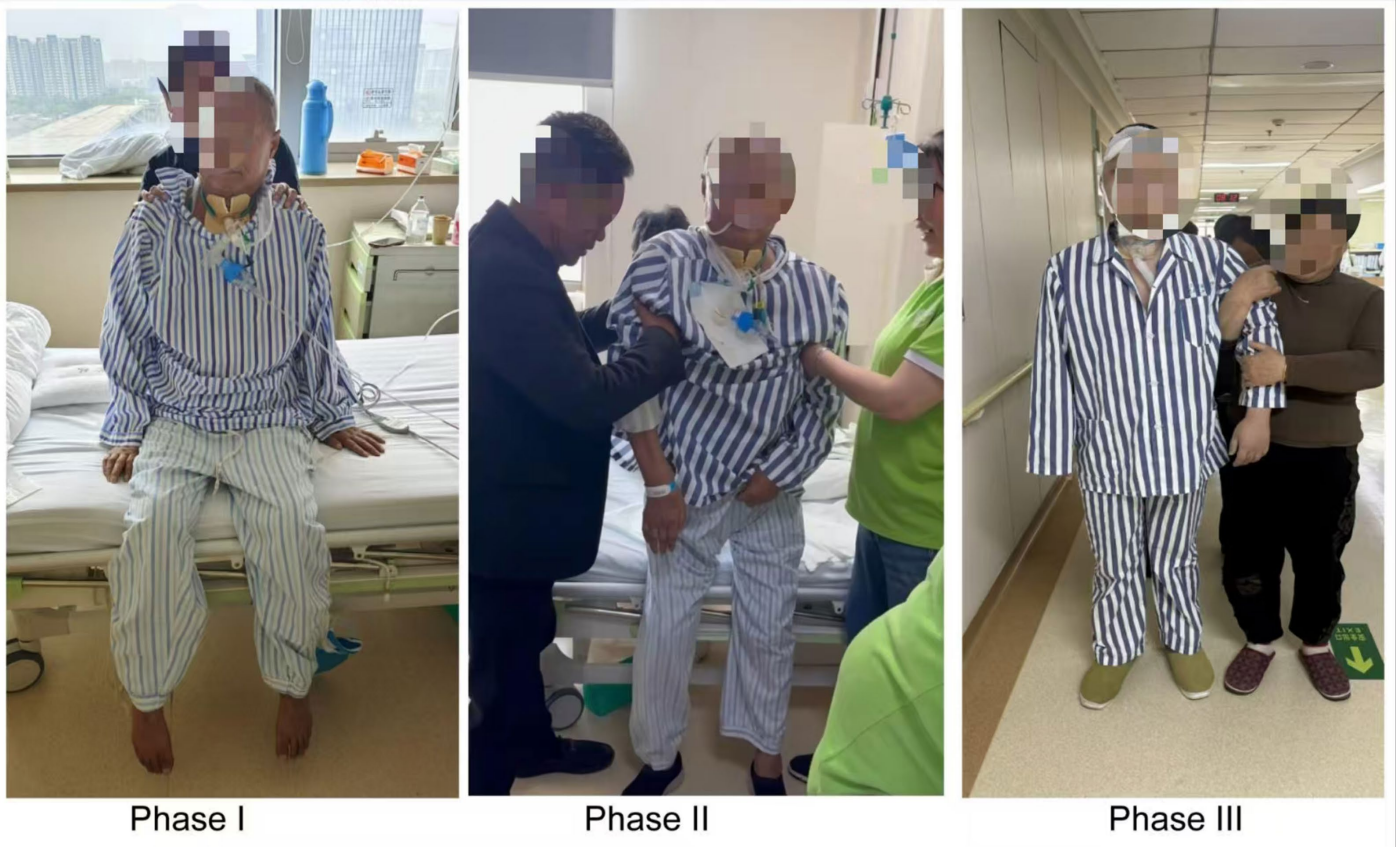
The early progressive ambulation intervention in neurosurgical patients undergoing tracheostomy.

The progressive ambulation group in this study implemented a standardized, phased mobilization protocol as an adjunct to conventional care. This evidence-based protocol incorporated periodic clinical assessments to determine appropriate activity progression, with rehabilitation phases carefully tailored to each patient’s baseline functional status and recovery trajectory. The intervention followed a physiologically-graded sequence beginning with basic bed-level activities (supine-to-sitting transitions and edge-of-bed sitting), progressing to upright positioning (sitting in a chair and static standing), and ultimately advancing to ambulation training. This three-phase progression system was individually calibrated through continuous physiological monitoring and functional assessment to optimize both safety and therapeutic efficacy. Protocol implementation followed a stratified approach: patients demonstrating limited muscular capacity (MRC grade <3) initiated with passive range-of-motion exercises and supported sitting activities; those achieving moderate strength (MRC grade 3–4) progressed to active-assisted movements and weight-bearing standing exercises; while patients meeting predefined safety criteria advanced to supervised ambulation training. Activity intensity was dynamically titrated based on real-time physiological parameters (including heart rate, oxygen saturation, and perceived exertion) to maintain an optimal therapeutic window while preventing both overexertion and inadequate stimulus. This precision dosing approach aimed to maximize neuromuscular adaptation while minimizing adverse event risks. The detailed phase division and operational procedures are shown in Table 1 and Figure 1.

**Table 1:** The protocol for early progressive ambulation in neurosurgical patients undergoing tracheostomy.

Phase	GCS:3–8	GCS:9–12	GCS:13–15
Phase One: Elevation of the Bed Head.	The head of the bed is routinely elevated to 30 degrees.	The head of the bed is routinely elevated to 30 degrees.	The head of the bed is routinely elevated to 30 degrees.
	The head of the bed is elevated to 60 degrees (1–3 h)	The head of the bed is elevated to 60 degrees (0.5–1 h)	The head of the bed is elevated to 60 degrees (20–30 min)
	The patient is assisted into a 90-degree sitting position by family members, with the back separated from the bed panel, maintaining a right angle to the bed surface (0.5 to 1 h per session, twice daily).	① 90-degree sitting position (with family assistance, the patient’s back is lifted off the bed panel to assume a sitting position at a 90-degree angle to the bed surface) (1 to 2 h per session, twice daily). ② Bedside sitting with assistance from two caregivers (as tolerated by the patient). ③ Bedside sitting with assistance from one caregiver (as tolerated by the patient).	① Bedside sitting with assistance from one caregiver (1.5 to 2.5 h per session, twice daily). ② Independent sitting position (as tolerated by the patient).
Progression criteria for next phase	/	The patient can tolerate the maximum activity level of the current phase without worsening of their condition, and is ready to progress to bedside standing.	
Phase Two: Bedside standing	/	① Bedside standing with assistance from two caregivers (starting from 1 min, gradually increasing duration based on the patient’s tolerance). ② Wall-supported standing with assistance from two caregivers (starting from 1 min, gradually increasing duration based on the patient’s tolerance).	① Bedside standing with assistance from two caregivers (starting from 1 min, gradually increasing duration based on the patient’s tolerance). ② Standing with assistance from one caregiver (starting from 1 min, gradually increasing duration based on the patient’s tolerance).
Progression criteria for next phase	/	The patient can tolerate the activities of the current phase without worsening of their condition and is ready to progress to ambulation.	
Phase Three: Ambulation.	/	Ambulation is initially performed with assistance from two caregivers, adjusted according to the patient’s tolerance, and gradually transitioned to walking with assistance from one caregiver.	Ambulation is performed with assistance from one caregiver, adjusted according to the patient’s tolerance, and gradually transitioned to independent walking.

1. Patients may wear monitoring devices, infusion pumps, nutritional pumps, or other equipment during the above activities. The use of such equipment should not hinder out-of-bed mobilization.

2. The out-of-bed mobilization plan should be adjusted according to the patient’s circadian rhythm, daily schedule, and lifestyle habits (e.g., sitting up in the morning, resting at noon, sitting up before meals, and changing positions 1 h after meals).

Suspension Criteria: ① Heart rate < 40 beats per minute or > 120 beats per minute; ② Respiratory rate < 5 breaths per minute or > 40 breaths per minute; ③ SpO2 < 88%; ④ Intracranial pressure > 25 mmHg; ⑤ Falls; ⑥ Catheter dislodgement; ⑦ Pain.

GCS, Glasgow Coma Scale.

To ensure the scientific rigor, validity, and efficient progression of this study, a MDT was established to provide interdisciplinary professional support and facilitate the integration of resources. The MDT consisted of five core members, each with distinct professional backgrounds and responsibilities: The team included two clinical nurses who played pivotal roles in the study. One nurse was responsible for the overall coordination of the project, including the implementation of the study protocol, coordination of the research process, and daily management of participants. The other nurse focused on data collection and analysis, systematically organizing, entering, and conducting preliminary analyses of the data generated during the study. This ensured the integrity and accuracy of the data, thereby providing a reliable basis for subsequent statistical analyses and interpretation of results. In addition to the nurses, the team comprised a neurosurgeon and a head nurse from the neurosurgical department. The neurosurgeon, leveraging extensive professional knowledge and clinical experience, provided medical guidance for the entire project, ensuring the scientific soundness and feasibility of the study protocol. This role also involved timely assessment and management of any medical issues that arose during the study. The head nurse was responsible for overseeing the project, ensuring strict adherence to the predetermined protocol, and maintaining the quality of nursing practices throughout the study. This role was crucial in safeguarding the well-being of participants and ensuring the smooth progression of the research. The team also included a rehabilitation physician, who was primarily responsible for the implementation and supervision of the intervention measures. During the progressive ambulation protocol, the rehabilitation physician developed personalized rehabilitation plans based on individual patient conditions and monitored the execution of the rehabilitation training to ensure its efficacy and safety. Additionally, the rehabilitation physician provided professional training and technical guidance to the nursing staff involved in the rehabilitation training, thereby enhancing the overall level of rehabilitation care within the team. Through the collaborative efforts of the MDT, each member contributed their unique expertise while working in concert with one another. This interdisciplinary cooperation formed an efficient and cohesive research framework, providing robust organizational support and professional backing for the successful conduct of this study.

In our study, the indications for initiating and discontinuing antibiotics were strictly adhered to established clinical guidelines and protocols (19, 20). Antibiotic therapy was initiated when there was a clinical suspicion of infection, which was subsequently confirmed through microbiological or radiological evidence. The duration of antibiotic use was tailored to the individual clinical course of each patient. In certain cases, extended courses of antibiotic therapy were deemed necessary due to the complexity of the infection, delayed clinical response, or the presence of resistant pathogens.

The following demographic and clinical information was collected for each patient: gender, age, body mass index (BMI), Glasgow Coma Scale (GCS) score at admission, history of hypertension, history of smoking, history of cerebral hemorrhage, and history of cerebral infarction. In this study, we collected several key outcome measures to comprehensively evaluate the impact of interventions on patients undergoing tracheotomy. The incidence of pulmonary infection was assessed based on diagnostic criteria. This included evaluating patients for fever (≥38.5°C), elevated leukocyte counts (≥10 × 10^9^/L), typical respiratory symptoms, auscultation findings, radiographic evidence of inflammation, and positive sputum cultures. Mortality rate was defined as the proportion of deaths attributable to pulmonary infections following tracheotomy, reflecting the severity of these infections. Antibiotic Use Density (AUD) was calculated to quantify antibiotic exposure, using the World Health Organization’s Defined Daily Dose (DDD) as a standardized measure. The formula used was AUD = Total DDD of antibiotics / Total patient-days × 100, providing insights into the appropriateness of antibiotic use and its potential impact on medical quality and patient safety. Additionally, the duration of antibiotic use was recorded to assess the overall exposure period, which is crucial for understanding antimicrobial stewardship practices. Tracheotomy duration was measured from the date of the procedure to successful decannulation, while the length of hospital stay was calculated from admission to discharge, both serving as indicators of the overall healthcare burden and patient recovery time. Finally, hospitalization costs were documented to reflect the economic impact of tracheotomy and associated complications on healthcare resources. These measures collectively provide a detailed assessment of clinical outcomes, resource utilization, and economic implications in patients undergoing tracheotomy.

Statistical analyses were conducted using SPSS version 25.0 to ensure the scientific rigor and accuracy of the results. Appropriate statistical methods were applied based on the type of data variables. For dichotomous variables, such as gender and the occurrence of pulmonary infection, descriptive statistics were presented as percentages (%). Between-group differences were analyzed using either the chi-square test (*χ*^2^ test) or Fisher’s exact test. The chi-square test was used for comparisons of dichotomous variables in larger sample sizes, while Fisher’s exact test was employed for smaller sample sizes to provide more precise statistical results, thereby ensuring the accuracy of analyses in scenarios with limited sample numbers. For continuous variables, including age, length of hospital stay, and duration of antibiotic use, descriptive statistics were reported as mean ± standard deviation. The mean reflects the central tendency of the data, while the standard deviation quantifies the dispersion of the data points. Between-group comparisons were performed using the independent samples t-test to determine significant differences in mean values between two groups. In all statistical analyses, a significance level of *p* < 0.05 was used to determine statistical significance.

## Results

**Figure 2:**
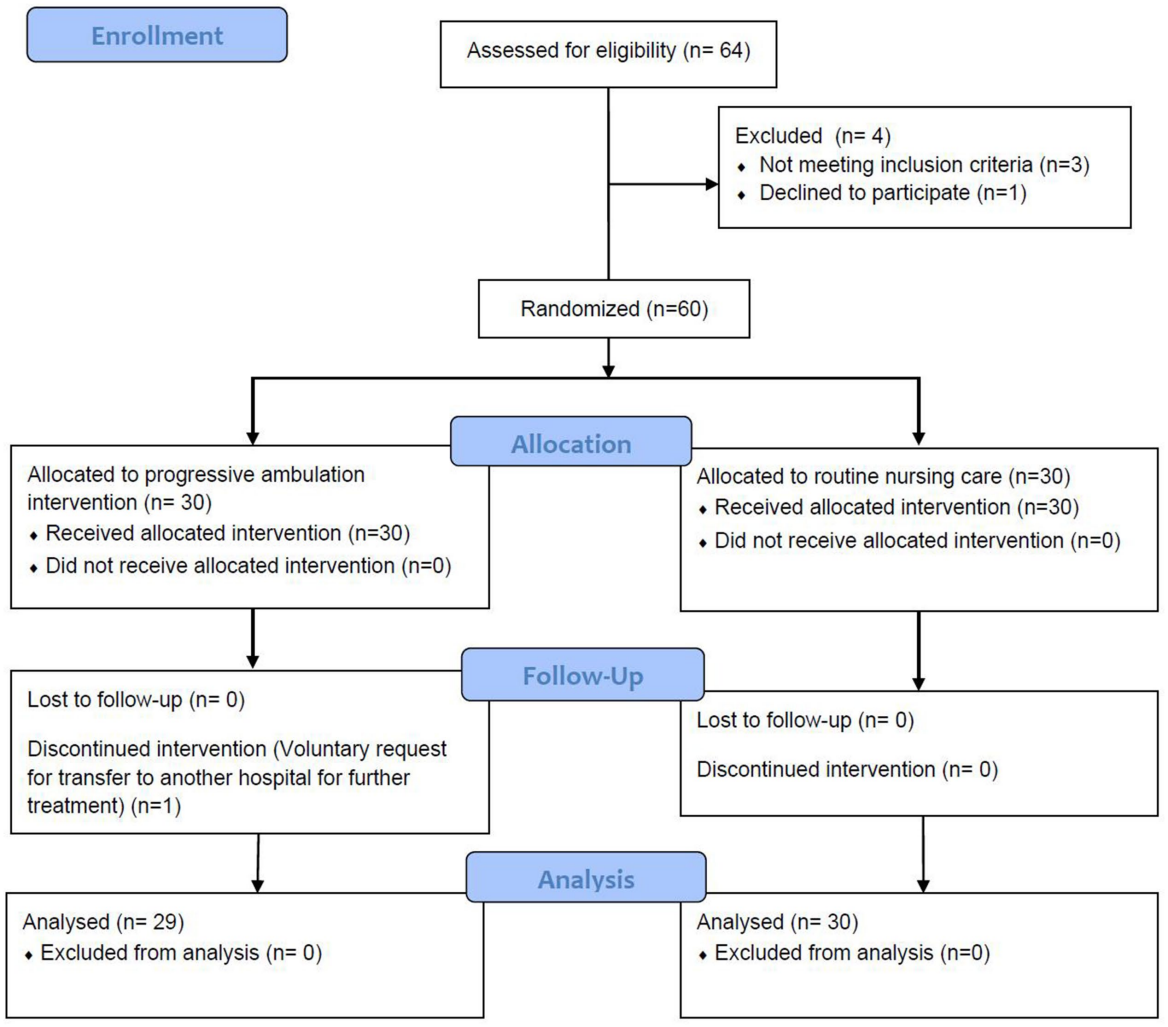
The flow diagram of patient inclusion.

As depicted in Figure 2, a total of 59 neurosurgical patients who underwent tracheostomy were ultimately included in the study. Of these, 29 patients received the progressive ambulation intervention, while 30 patients received standard routine nursing care. As presented in Table 2, there were no statistical differences in the gender, age, BMI, GCS score at admission, disease diagnosis, history of hypertension, history of smoking, history of cerebral hemorrhage, history of cerebral infarction and aspiration occurred on admission and use of sedative drugs between ambulation group and control group (all *p* > 0.05).

**Table 2:** Characteristics of included neurosurgical patients undergoing tracheostomy.

Characteristics	Ambulation group (*n* = 29)	Control group (*n* = 30)	*t*/*F*	*p*
Male/female	20/9	22/8	0.512	0.184
Age (y)	62.05 ± 10.74	61.48 ± 10.99	5.276	0.082
BMI	21.69 ± 2.13	21.80 ± 2.35	2.344	0.102
GCS at admission	8.77 ± 3.24	8.81 ± 3.31	1.746	0.068
Disease diagnosis			1.628	0.145
Ischemic stroke	19(65.52%)	21(70.00%)		
Hemorrhagic stroke	10(34.48%)	9(30.00%)		
History of hypertension	17(58.62%)	18(60.00%)	1.328	0.114
History of Smoking	9(31.03%)	10(33.33%)	1.274	0.092
History of cerebral hemorrhage	2(6.90%)	2(6.67%)	1.558	0.104
History of cerebral infarction	2(6.90%)	3(10.00%)	1.298	0.087
Aspiration occurred on admission	3(10.34%)	2(6.67%)	2.041	0.116
Use of sedative drugs	21(72.41%)	22(73.33%)	1.845	0.077

BMI, Body Mass Index; GCS, Glasgow Coma Scale.

As shown in Table 3, there was statistical difference in the incidence of pulmonary infection between ambulation group and control group (*p* = 0.02), the incidence of pulmonary infection of ambulation group was significantly lower than that of control group. There was no statistical difference in the mortality between ambulation group and control group.

**Table 3:** Comparison of the incidence of pulmonary infection and mortality rate between the two groups.

Variables	Ambulation group (n = 29)	Control group (n = 30)	F	p
Pulmonary infection	6(20.69%)	16(53.33%)	2.104	0.002
Mortality	0(0%)	0(0%)		1.00

In the ambulation group, both the AUD and the duration of antibiotic use were significantly lower than those in the control group. The differences between the two groups were statistically significant (all *p* < 0.05). Detailed results are presented in Table 4.

**Table 4:** Comparison of antibiotic use density (AUD) and duration of antibiotic use between the two patient groups.

Variables	Ambulation group (*n* = 29)	Control group (*n* = 30)	*t*	*p*
AUD (DDDs/100 patient-days)	56.70 ± 14.23	71.42 ± 16.08	9.553	0.040
Duration of antibiotic use (d)	25.85 ± 10.51	30.29 ± 12.76	3.239	0.016

AUD, Antibiotic Use Density.

As shown in Table 5, compared with the control group, the ambulation group exhibited significantly shorter durations of tracheostomy, hospital stay, and lower hospitalization costs. The differences between the two groups were statistically significant (all *p* < 0.05).

**Table 5:** Comparison of tracheostomy duration, length of hospital stay and hospitalization costs between the two groups.

Variables	Ambulation group (*n* = 29)	Control group (*n* = 30)	*t*	*p*
Tracheostomy duration (d)	21.05 ± 10.85	30.66 ± 14.26	3.905	0.014
Length of hospital stay (d)	37.32 ± 18.24	44.24 ± 20.64	5.018	0.022
Hospitalization costs (CNY)	98963.79 ± 2100.63	11647.42 ± 2205.26	44.599	0.038

CNY, China Yuan.

## Discussion

The concept of progressive ambulation can be traced back to the late 19th century, when early studies demonstrated that mobilization soon after surgery could significantly reduce postoperative hospital stays (21). By the late 20th century, as understanding of the complications associated with prolonged sedation and mechanical ventilation deepened, early mobilization began to be incorporated into clinical practice in ICUs. It has been reported that daily interruption of sedation could significantly shorten the duration of mechanical ventilation and ICU length of stay (22). Early mobilization could improve respiratory function, reduce the incidence of intensive care unit-acquired weakness (ICU-AW), and decrease the duration of mechanical ventilation (23).

In recent years, the application of progressive ambulation in ICUs has gradually increased. For example, Bailey et al. documented 1,449 instances of early mobilization interventions among 103 patients, with 53% of mechanically ventilated patients engaging in out-of-bed activities (24). Another study demonstrated that ICU patients who underwent early mobilization interventions had significantly shorter hospital stays and better functional recovery (25). However, despite the theoretical advantages of early mobilization, its widespread implementation in clinical practice remains challenging. Many ICUs initiate physical therapy only after patients are extubated, whereas early mobilization should ideally begin within 48 h of initiating mechanical ventilation and continue throughout the ICU stay (26). The implementation of progressive ambulation requires the collaboration of a multidisciplinary team, including physicians, nurses, physical therapists, and respiratory therapists (27). These studies underscore the critical role of multidisciplinary collaboration in the successful implementation of early mobilization.

Progressive ambulation significantly reduces the incidence of pulmonary infection, aligning with the recognized benefits of early mobilization in ICUs. This reduction is achieved through improvements in respiratory function and a decrease in complications associated with prolonged bed rest (28). Early mobilization enhances oxygenation and reduces the incidence of atelectasis and pleural effusion, which are likely key mechanisms contributing to its effectiveness in lowering pulmonary infection rates (29). The benefits of early mobilization can be attributed to multiple factors: it promotes early out-of-bed activities, thereby reducing respiratory stasis and sputum accumulation, and decreasing the risk of respiratory tract infections (30). It also strengthens respiratory muscles, improves oxygenation, and enhances spontaneous breathing capacity, further reducing pulmonary complications (31). Additionally, early mobilization may improve systemic circulation and boost immune function, both of which contribute to a lower risk of infection (32). Collectively, these mechanisms underpin the significant clinical advantages of progressive ambulation in reducing pulmonary infection incidence.

Progressive ambulation significantly reduces AUD and the duration of antibiotic use, an effect that may be attributed to multiple factors. Progressive ambulation enhances systemic circulation and immune function, thereby reducing the demand for antibiotics driven by infections. This type of activity strengthens patients’ overall physical capacity and promotes recovery, which in turn lowers the incidence and severity of infections (33). Additionally, reducing antibiotic use not only helps mitigate the development of antimicrobial resistance but also decreases healthcare costs (34). Antimicrobial resistance represents one of the major challenges to global public health, and reducing unnecessary antibiotic use is a key strategy in addressing this issue (35). Therefore, progressive ambulation is not only an effective rehabilitation intervention but also a potential strategy to reduce inappropriate antibiotic use. This finding suggests that the implementation of early mobilization should be prioritized in clinical practice to optimize patient outcomes and reduce the consumption of healthcare resources.

The results of this study demonstrate that progressive ambulation significantly reduces the duration of tracheostomy and length of hospital stay. These findings are consistent with the recognized benefits of early mobilization in improving patients’ functional status and accelerating recovery (36). By promoting early out-of-bed activities and reducing bed rest time, early mobilization mitigates complications associated with prolonged immobility, such as deep vein thrombosis and pressure ulcers (37). Additionally, early mobilization has been shown to improve patients’ psychological states and reduce the incidence of delirium, which may further enhance the recovery process (38). Progressive ambulation also significantly lowers hospitalization costs, likely due to the reduced length of stay, decreased incidence of complications, and diminished use of antibiotics (39). Moreover, early mobilization reduces reliance on high-cost medical resources, such as mechanical ventilation and intensive care (40). Therefore, progressive ambulation not only improves clinical outcomes for patients but also represents a potential strategy for reducing healthcare costs.

Based on the current findings, we advocate for the clinical adoption of progressive ambulation, particularly in neurosurgical patients with tracheostomy, while emphasizing the need for a structured implementation framework. A multidisciplinary team (MDT) approach involving physicians, nurses, physical therapists, and dietitians is essential to develop and execute personalized rehabilitation plans (41), complemented by systematic training programs to enhance healthcare providers’ competency in early mobilization protocols (42). Future research should focus on identifying optimal patient populations for this intervention, establishing standardized protocols adaptable to diverse clinical settings (43), and evaluating long-term outcomes. Importantly, implementation strategies must address safety concerns through technological innovations in assistive devices and rigorous monitoring protocols to mitigate risks such as equipment dislodgement and patient falls (44). Despite existing evidence supporting early mobilization, its full integration into routine practice requires both refinement of clinical protocols and development of supportive infrastructure. Through these coordinated efforts, progressive ambulation can be effectively established as a fundamental component of rehabilitation programs, ultimately improving patient outcomes while maintaining the highest safety standards. The successful translation of these findings into clinical practice will depend on continued interdisciplinary collaboration and ongoing evaluation of implementation outcomes.

This RCT has several limitations that should be acknowledged and addressed in future research. Firstly, the study was conducted as a single-center investigation with a relatively small sample size. This design choice may limit the generalizability of the findings to broader populations, as it could introduce biases related to specific population characteristics or geographic factors. Secondly, due to constraints in human resources and funding, it was not feasible to blind either the intervention providers or the outcome assessors. This lack of blinding may have potentially influenced the objectivity of the study results, thereby affecting the interpretation of the findings. Future research should consider adopting a multicenter design to increase the sample size and enhance the representativeness of the study population. Additionally, incorporating blinding methods, where feasible, would significantly enhance the reliability and generalizability of the study findings. Such improvements would contribute to a more robust evaluation of the intervention’s efficacy and safety.

## Conclusion

In conclusion, this study find that progressive ambulation offers significant clinical benefits for neurosurgical patients undergoing tracheostomy. Specifically, it reduces the incidence of pulmonary infection, AUD, and duration of antibiotic use. Additionally, it shortens the duration of tracheostomy and length of hospital stay, while also lowering hospitalization costs. These findings suggest that progressive ambulation not only enhances clinical outcomes but also optimizes the efficiency of healthcare resource utilization. Given these results, we recommend incorporating progressive ambulation into the clinical management of neurosurgical patients with tracheostomy. To ensure its safety and efficacy, we advise forming a MDT comprising physicians, nurses, physical therapists, and dietitians. This team can collaboratively develop and implement personalized rehabilitation plans tailored to individual patient needs. Future research should further explore the applicability and long-term effects of progressive ambulation across diverse patient populations. This will help optimize clinical practice and validate the broader implementation of this intervention. Through these efforts, progressive ambulation has the potential to become a key strategy for improving outcomes in neurosurgical patients with tracheostomy.

## Data Availability

The original contributions presented in the study are included in the article/supplementary material, further inquiries can be directed to the corresponding author.
